# Genistein Partly Eases Aging and Estropause-Induced Primary Cortical Neuronal Changes in Rats

**DOI:** 10.1371/journal.pone.0089819

**Published:** 2014-02-25

**Authors:** Tsyr-Jiuan Wang, Jeng-Rung Chen, Wen-Jay Wang, Yueh-Jan Wang, Guo-Fang Tseng

**Affiliations:** 1 Department of Nursing, National Taichung University of Science and Technology, Taichung, Taiwan; 2 Department of Veterinary Medicine, College of Veterinary Medicine, National Chung-Hsing University, Taichung, Taiwan; 3 Department of Anatomy, College of Medicine, Tzu-Chi University, Hualien, Taiwan; University of Nebraska Medical Center, United States of America

## Abstract

Gonadal hormones can modulate brain morphology and behavior. Recent studies have shown that hypogonadism could result in cortical function deficits. To this end, hormone therapy has been used to ease associated symptoms but the risk may outweigh the benefits. Here we explored whether genistein, a phytoestrogen, is effective in restoring the cognitive and central neuronal changes in late middle age and surgically estropause female rats. Both animal groups showed poorer spatial learning than young adults. The dendritic arbors and spines of the somatosensory cortical and CA1 hippocampal pyramidal neurons were revealed with intracellular dye injection and analyzed. The results showed that dendritic spines on these neurons were significantly decreased. Remarkably, genistein treatment rescued spatial learning deficits and restored the spine density on all neurons in the surgically estropause young females. In late middle age females, genistein was as effective as estradiol in restoring spines; however, the recovery was less thorough than on young OHE rats. Neither genistein nor estradiol rectified the shortened dendritic arbors of the aging cortical pyramidal neurons suggesting that dendritic arbors and spines are differently modulated. Thus, genistein could work at central level to restore excitatory connectivity and appears to be potent alternative to estradiol for easing aging and menopausal syndromes.

## Introduction

Dendrites, the primary sites of synaptic inputs in central neurons, are dynamic structures whose arbors and spines change in normal situation upon environmental changes [1], hormonal fluctuation [2] and aging [3]–[6], and in addition in response to injuries [7]–[10], and diseases [11]. Gonadal or sex hormones had been reported to alter the dendrites of hippocampal, anterior cingulate and prefrontal cortical neurons [12]–[15]. Recently we demonstrated that in normal adult female rats, dendritic spines on primary somatosensory cortical neurons changed cyclically during the relatively fast estrous cycle [2]. Ovarihysterectomy (OHE) broke this cycle and substantially reduced the dendritic spines on these neurons which could be reversed following exogenous gonadal hormone treatment [2]. As expected, dendritic spines on somatosensory cortical pyramidal neurons were reduced following normal aging [6]. Intuitively, sex hormone supplement appears to be the way to ease hypogonadism associated symptoms including improving cortical functions in natural aging or under diseases [16]–[17]. Phytoestrogens are plant compounds that exert estradiol-like physiological effects in both humans and rats [18], [19]. They consequently received a great deal of attention as alternatives of estradiol for the potential preventive roles against some of today’s most prevalent hypogonadism-associated chronic diseases, such as cardiovascular disease, osteoporosis and hormone-related cancers [20]–[22]. These estrogen-like molecules are found in many plants and are especially abundant in soy products [18]. The particular molecules of interest are isoflavones, which are found in abundance in soybeans and the derived foods, such as tofu, miso, and others. Of the several soybean isoflavones, genistein is shown to be the most effective in human and animal experiments [18], [23], [24]. In light of this, we used female rats as model and investigated whether genistein supplement can reverse the behavioral and morphological alterations found following OHE surgery and normal aging. To characterize brain neuronal morphological alterations, the dendritic arbors and spines of the pyramidal neurons in the primary somatosensory cortex and hippocampus were revealed with intracellular dye injection. Dendritic arbors were analyzed following 3-dimensional reconstruction.

## Materials and Methods

### Animals

All experimental protocols were ethically approved and carried out in accordance with the National Chung-Hsing University’s Intramural Animal Care and Use Committee under project license 98-82. All efforts were taken to minimize animal suffering during and following surgery. Thirty 8–12-week (nulliparous) and 15 1.5 year-old (breeders retired at 5–7 month of age), correspond to young and late middle age [25] adult female Sprague-Dawley rats (BioLasco, Ilan, Taiwan) were studied. Animals were housed in temperature (24±1°C), humidity (60% ±5%) and light-controlled (light on at 06∶00 h and off at 18∶00 h) environment. They were fed with phytoestrogen-free diet (LabDiet, 5K96) *ad libitum* considering that soy products are the major protein source in all commercially available rodent diets (approximately 200 to 600 µg/g). Six diestrous young adult females, confirmed with vaginal smear, served as young adult control. The rest of the young adult female rats, 24 of them, were deeply anesthetized with ketamine and xylazine (8 mg ketamine and 1 mg xylazine/100 g body weight) and subjected to OHE as described before [2]. They were then divided into two groups, n = 12 each, to survive for 2 (OHE-2W) and 8 weeks (OHE-8W), respectively. Half of each of the OHE-2W and OHE-8W group animals were treated with 5 consecutive days of subcutaneous genistein injection (Sigma-Aldrich, Saint Louis, MO, 5 mg/kg, in Sesame Oil), and the other half with vehicle (1 ml/kg, Sesame oil) starting 5 days before scheduled to be sacrificed. Subcutaneous administration was chosen over oral to avoid variation in the amount of ingestion and/or gut absorption between animals. Insulin syringe (29G needle) was used for the injection to minimize stress. All animals were run on Morris water maze task (please see below) starting 3 days before scheduled to be sacrificed, i.e., starting on the third day of genistein/vehicle treatment in the experimental groups.

In the late middle age animal experiment, the 15 rats were divided initially into 4 groups: group 1 (n = 4) was free from any treatment (late middle age); group 2 received vehicle injection (sesame oil) (n = 3, late middle age+V); group 3 received genistein injection (5 mg/kg, in Sesame Oil) (n = 4, late middle age+GS) for 5 consecutive days immediately before scheduled to be sacrificed; group 4 (n = 4, late middle age+E2) received subcutaneous estradiol pellet implantation (0.5 mg/pellet, 21-day release, Innovative Research of America, USA) 14 days before the scheduled sacrifice date following our previous protocol [2]. All animals were run on Morris water maze task starting 3 days before scheduled to be sacrificed.

### Morris Water Maze Task

We followed previous protocols [26] to evaluate the learning and memory performance of the animals. Animal performance was recorded with a video camera for subsequent analysis of the swimming path and speed. The maze apparatus consisted of a circular pool 200 cm in diameter and 60 cm deep. The pool was filled with water at approximately 23°C to a height of 50 cm. A transparent platform (diameter 15 cm) is placed at a constant position 2–3 cm below the surface of the water. The visual cues arrayed around the room were available for the rats to learn the location of the hidden platform. All rats of each group were tested for 3 consecutive days with two trials per day. Rats were randomly placed into different quadrant of the pool in each trail. Rats were allowed to remain on the platform for 20 s if escaped within 180 s, or alternatively placed on the platform and remained there for 20 s if failed to locate the underwater platform within 180 s. A recovery period of 10 minutes was allowed between the two trials. The escape times of the two trials conducted each day were recorded and averaged.

### Intracellular Dye Injection and Immunoconversion of the Injected Dye

Animals processed for fixed tissue intracellular dye injection were deeply anesthetized as described above and perfused with a fixative containing 2% paraformaldehyde in 0.1 M phosphate buffer (PB), pH 7.3, for 30 min. The brain was immediately removed and sectioned with vibratome into 350-µm-thick coronal slices. For dye injection, brain slices were first treated with 0.1 M PB containing 10^−7^ M 4′, 6-diamidino-2-phenyl-indole (DAPI; Sigma-Aldrich, St. Louis, MO) for 30 minutes to make cell nuclei fluoresced blue under the filter set that visualized the yellow fluorescence of intracellular dye Lucifer yellow (LY, Sigma-Aldrich) [7]. This permitted the selection of cells for dye injection. The brain slice was then placed in a shallow chamber on the stage of a fixed-stage, fluorescence microscope (Olympus BX51) and covered with 0.1 M PB. Intracellular micropipette filled with 4% LY in water was positioned with a three-axial hydraulic micromanipulator (Narishige, Tokyo, Japan) to impale selected neurons for dye injection. The injection current was generated by an intracellular amplifier (Axoclamp–IIB). At the end of injection, the slice was rinsed in 0.1 M PB and postfixed in 4% paraformaldehyde in 0.1 M PB before cryoprotection and subsequently sectioned into 60-µm-thick serial sections for immunoconversion.

To convert the fluorescence dye into nonfading material, the above tissue sections were first incubated with 1% H_2_O_2_ in 0.1 M PB for 30 min to remove endogenous peroxidase activity and then incubated in PBS containing 2% Bovine Serum Albumin (Sigma-Aldrich) and 1% Triton X-100. Sections were then treated with biotinylated rabbit anti-LY (1∶200; Molecular Probes, Eugene, OR) in PBS for 18 hours at 4°C and then with standard avidin-biotin HRP reagent (Vector, Burlingame, CA) for 3 hours at room temperature. They were then reacted with 0.05% 3-3′-diaminobenzidine tetrahydrochloride (DAB, Sigma-Aldrich) and 0.01% H_2_O_2_ in 0.05 M Tris buffer. Reacted sections were mounted onto slides for 3-dimensional reconstruction.

### Blood Estrogen Measurement

Blood samples (1.5 ml) were collected via the inferior vena cava when sacrificing the animals (9∶00 in the morning). The sample was centrifuged (3000×*g*, 15 min, 4°C) before measurement. Level of estrogen was assayed with an automated ADVIA Centaur® Immunoassay System (Bayer, Germany) with ADVIA Centaur E26 ReadyPack (02531842) commissioned by a clinical laboratory (UM Clinical Laboratory, Taichung, Taiwan).

### 3-dimensional Reconstruction and Data Analysis

To study the changes of dendritic arbor and length, we followed our previous protocol [2] to reconstruct the dendritic arbor of each neurons from the serial sections of the brain slice in which it was dye-injected (please see supplementary figure S1 for details). The complete dendritic arbors of 5 layer III and 5 layer V neurons of the somatosensory cortex of each rat were reconstructed 3-dimensionally with Neurolucida (MicroBrightField, Williston, VT). The mean of the dendritic lengths of the 5 neurons of each animal is the dendritic length of the particular type of neuron of the animal. To analyze dendritic spine density, we analyzed 5 representative CA1 hippocampal and 5 layer III and 5 layer V somatosensory cortical pyramidal neurons from each animal. Dendrites of the layer III and layer V cortical pyramidal neurons were distinguished into proximal and distal segments of the apical and basal dendrites following the criteria described before [7], [26]. Briefly, for layer III pyramidal neurons, proximal and distal basal dendrites were defined as the segments 25–75 *µ*m (around the first to second branch) and 100–150 *µ*m (around the last one or two branches) from the soma, respectively. For the relatively large layer V pyramidal neurons, proximal and distal basal dendrites were defined as the segments 50–100 *µ*m (around the first to second branch) and 150–200 *µ*m (around the last one or two branches) from where they originate from the soma, respectively. Proximal apical dendrites were the first or second branch of the apical trunk, and distal apical dendrites were the terminal dendrites after the last branch point in both layer III and V pyramidal neurons. For hippocampal CA1 pyramidal neurons, basal dendrites were defined as those in the stratum oriens while apical dendrites were on the other side of the cell body layer with the proximal segment in the stratum radiatum and distal segment in the stratum lacunosum-moleculare as the criteria described before [26]. Six random segments of each category of dendrites were sampled in each neuron and the mean represents the spine density of the particular segment of the neuron. The means of dendritic segment of the 5 neurons studied in each animal were then averaged to derive the spine density of the particular dendritic segment of the type of neuron of each animal. All data was expressed as mean ± SE. N represents the number of animals. Morris water maze data during the 3 days of tests and between groups were compared using repeated measures ANOVA test, followed by S-N-Ks’ post hoc. Data for dendritic structures were evaluated with one-way ANOVA followed by the Newman-Keuls test. Differences were considered statistically significant at p<0.05.

## Results

Blood estrogen analysis showed that the level of estrogen in all young adult OHE animals was below detectable whether they were treated with genistein or vehicle. This and the results of vaginal smear confirmed that all OHE young adult rats studied were in the state of persistent anestrus [26]. As expected, all late middle age females used in the present study also had low blood estrogen level. Genistein or vehicle treatment failed to alter the estrogen level while two weeks of estrogen pellet implantation increased it dramatically (Table 1).

**Table 1:** Blood estrogen level (pg/ml).

	Young adult female					Late middle age female			
	Normal	OHE-2 weeks		OHE-8 weeks					
Treatment	–	vehicle	genistein	vehicle	genistein	–	vehicle	genistein	estradiol
Estrogen level	20.9±3.9	ND	ND	ND	ND	4.8±1.9*	3.5±1.5*	4.5±1*	115.5±16.4* #
	(n = 6)	(n = 6)	(n = 6)	(n = 6)	(n = 6)	(n = 4)	(n = 3)	(n = 4)	(n = 4)

Normal young adult females were confirmed at diestrus with vaginal smear. The vehicle of genistein treatment was sesame oil. Values are means ± SE. ND, non-detectable;

*p<0.05 between the marked and young normal adult;

# p<0.05 between the marked and the late middle age rats, One-way ANOVA followed by S-N-K test.

### Effects of OHE and Subsequent Genistein Treatment on Young Adult Rats

**Figure 1:**
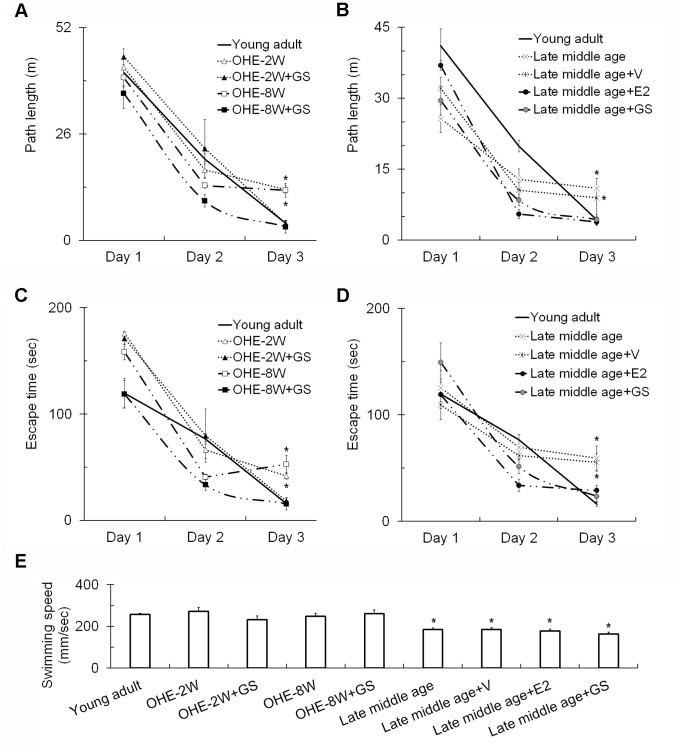
The effects of 5-day genistein treatment on spatial learning memory evaluated with Morris water maze task. The swimming distance (A and B) and escape latency (C and D) of the experimental animals to find the platform during the 3 consecutive days of Morris water maze task was plotted. The average swimming speed of each animal was shown in E. Young adult: young adult female rats; OHE-2W: OHE and survived for 2 weeks and vehicle-treated; OHE-2W+GS: OHE-2W females treated with genistein; OHE-8W: OHE for 8 weeks and vehicle-treated; OHE-8W+GS: OHE-8W females treated with genistein; late middle age: late middle age females without vehicle treatment; late middle age+V: late middle age females treated with vehicle; late middle age+GS: late middle age females treated with genistein; late middle age+E2: late middle age females treated with estradiol. *, p<0.05 between the marked and young adult, two-way ANOVA followed by S-N-K test. Vertical bar represents standard error of the mean.

To assess the spatial learning and memory, rats were tested for 3 consecutive days with Morris water maze task. Differences in swimming distance and escape latency between days and groups were analyzed with 2-way repeated measures ANOVA. On the first day, no difference in swimming distance was found between groups (F = 16.16, p = 0.17). The swimming distance shortened gradually over the 3 days of test in all groups (Fig. 1A and B), indicating gradual spatial memory acquisition in all animals. Significant differences between different days of test (F = 207.9, p<0.001) and among treatment groups (F = 13.34, p<0.001) were revealed following 2-way ANOVA. There were also significant interaction between day of test and treatments (F = 5.95, p<0.001). On the third day of training, the swimming distances of genistein-treated OHE rats (OHE-2w+GS, OHE-8w+GS) improved significantly to comparable to that of the control group (Fig. 1A, F = 14.73, p<0.05 genistein-treated OHE versus OHE rats). A similar trend of changes in escape time was observed in these rats. The escape time reduced gradually over the test period in all groups (Fig. 1C and D). Two-way ANOVA showed significant differences between the day of test (F = 250.75, p<0.001), and among treatment groups (F = 5.53, p<0.001) with a significant interaction between day of test and treatments (F = 3.763, p<0.001). On the third day of training, the escape latencies of the genistein-treated groups (OHE-2w+GS, OHE-8w+GS) became significantly shorter than that of the corresponding OHE rats (Fig. 1C, F = 11.65, p<0.05) and were no different from that of the control group. There was no difference in swimming speed between control, OHE and OHE+GS groups on the third test day (Fig. 1E, F = 2.1, p = 0.11). Genistein treatment significantly improved OHE animals’ performance to a level comparable to that of the young adult (Fig. 1A, and C) without affecting their motor ability as shown in the similarity in swimming speed (Fig. 1E). In addition, the plots in figure 1A and C show that prolonged survival, up to 8 weeks following OHE, did not compromise animal’s performance further as 2 and 8-week OHE rats showed comparable performance.

**Figure 2:**
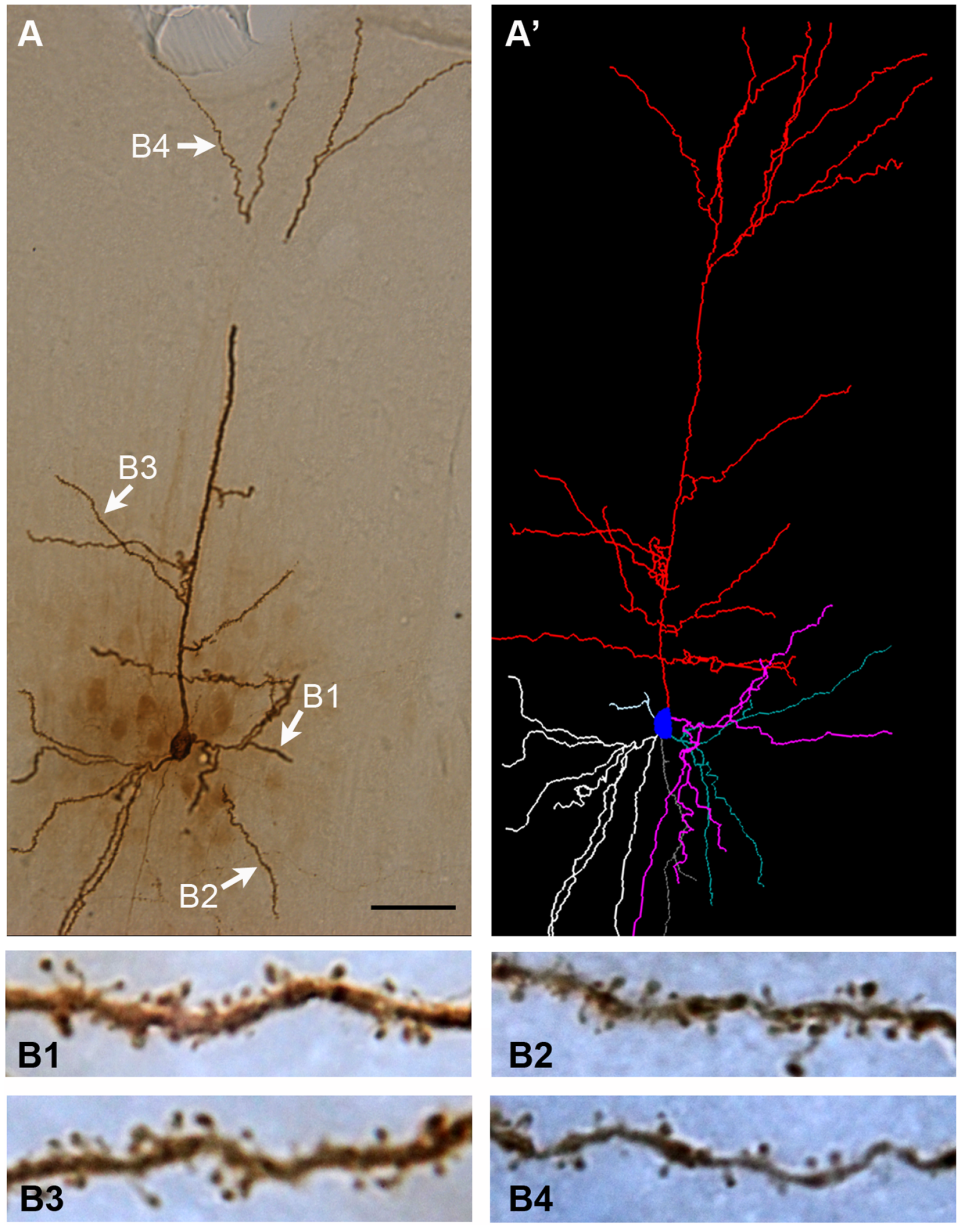
A representative layer III pyramidal neuron of the primary somatosensory cortex (control animal) revealed with intracellular dye injection. A is a low-power picture of the neuron from one of the 60-µm-thick sections of the brain slice in which the cell was filled. A’ was the dendritic arbor of the neuron reconstructed 3-dimensionally from all serial sections of the injected brain slice with computer software. The dendritic segments (B1–B4) indicated in A were illustrated at higher magnification at the bottom. Bar = 50 µm in A and A’ and 5 µm for B1∼B4.

With fixed tissue intracellular dye injection, we were able to examine the dendritic arbors of the studied neurons. Figure 2 illustrates the overall shape of a representative LY-filled layer III pyramidal neuron from a young adult animal. The photograph in figure 2A was taken from one of the 60-µm-thick sections of the injected slice. It demonstrates that the majority of the processes of a relatively large pyramidal neuron could be contained within a section if the slice was cut with the brain properly oriented. This enabled us to reconstruct the dendritic arbor of each neuron from the set of serial sections obtained from the brain slice in which the neuron was injected (Fig. 2A’; Fig. S1). Higher magnification views of segments of the proximal and distal basal and apical dendrites of the neuron (Fig. 2B1–4) showed that dendritic spines could be readily distinguished for subsequent analysis.

**Figure 3:**
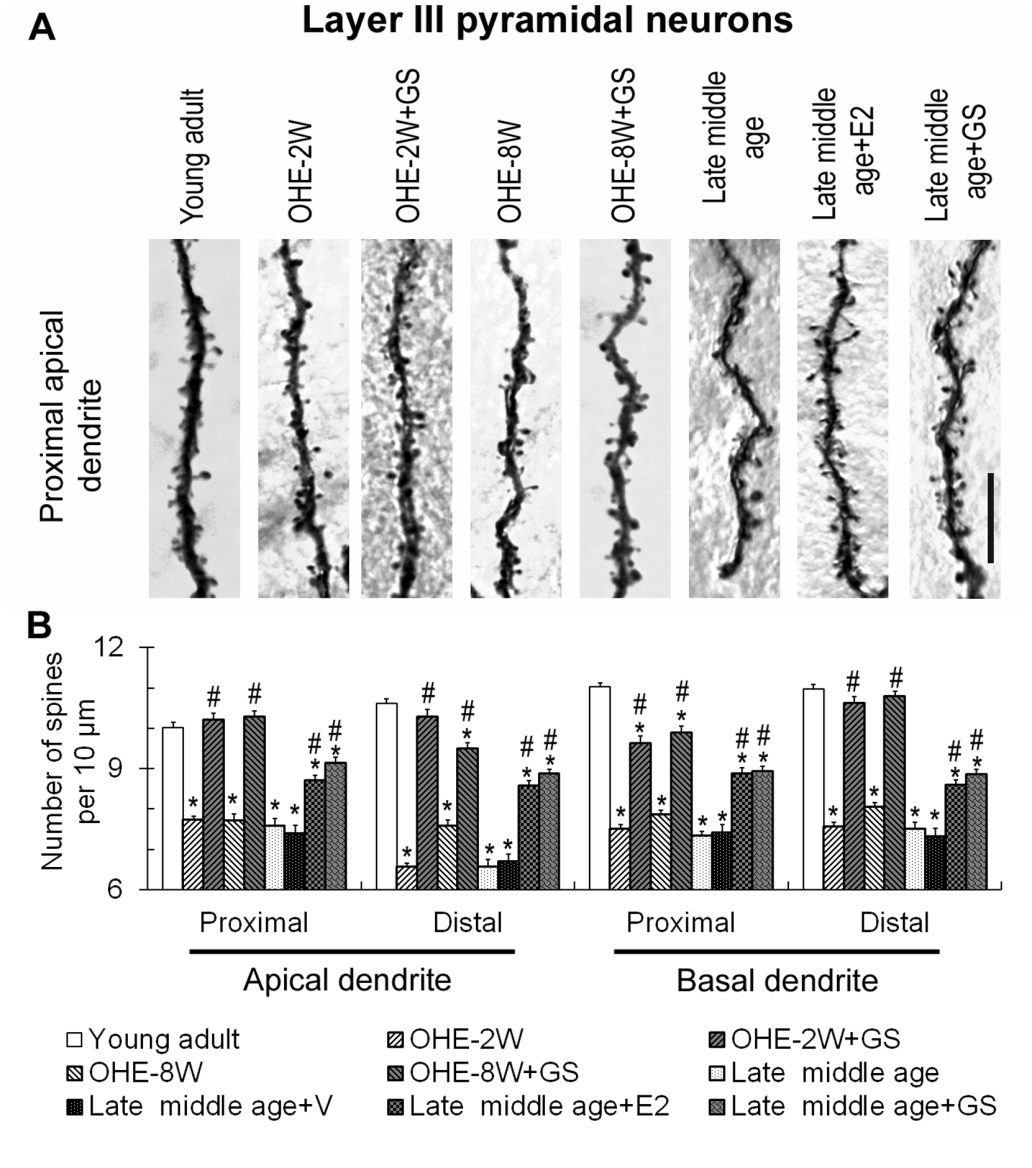
Changes in the dendritic spines of somatosensory cortical layer III pyramidal neurons in OHE and late middle age females and following treatments with gensitein (GS) and estradiol (E2). A shows the micrograph of a representative segment of the proximal apical dendrite of a neuron of the young adult, OHE-2W, OHE-2W+GS, OHE-8W, OHE-8W+GS, late middle age, late middle age+GS and late middle age+E2 group, respectively. Spine densities of these animal groups were plotted in B. Please see legend of figure 1 for abbreviations. *, p<0.05 between the marked and young adult; #, p<0.05 between the marked (GS or E2-treated) and its corresponding control, one-way ANOVA followed by Newman-Keuls test. Bar = 10 µm for all.

**Figure 4:**
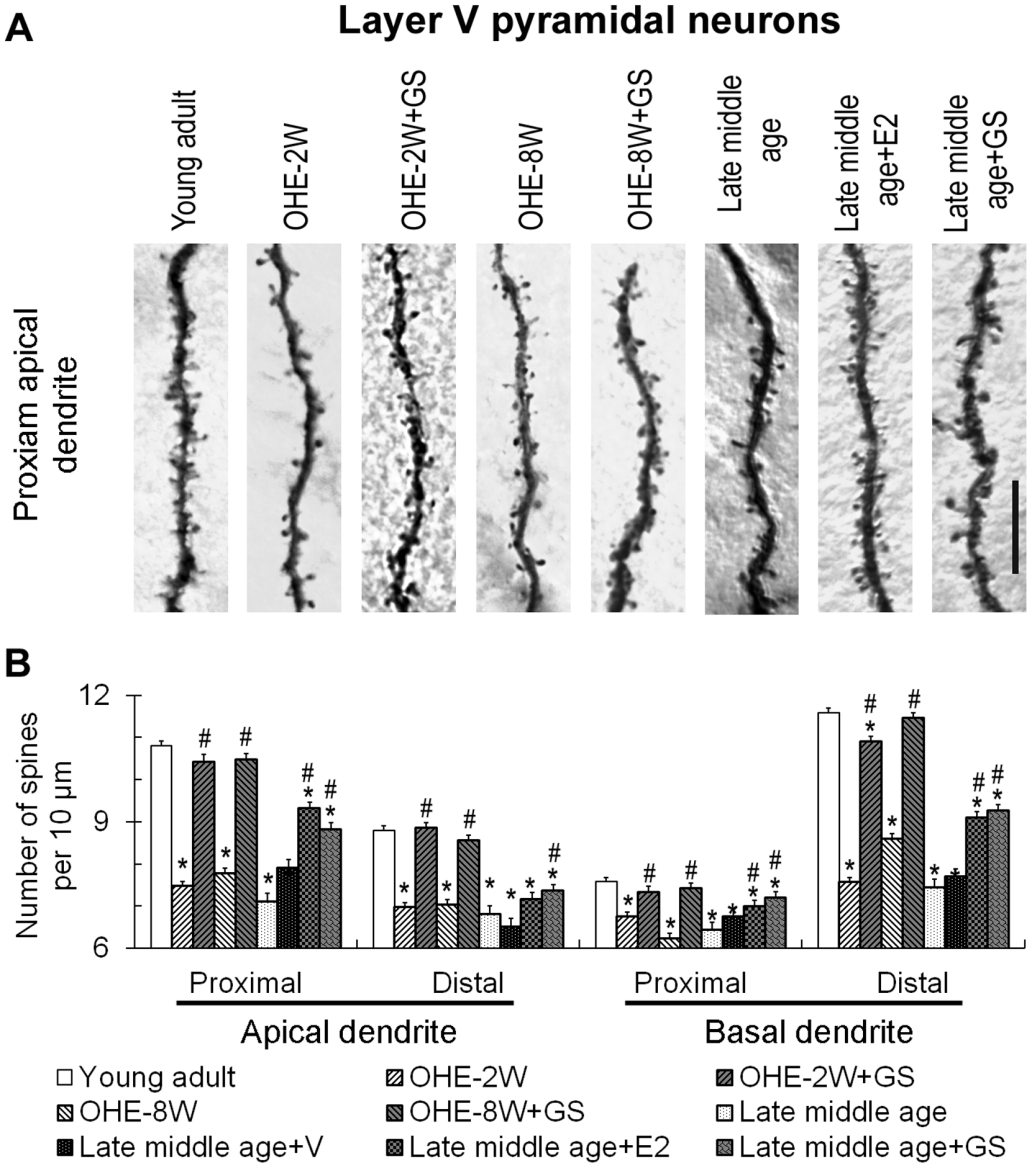
Changes in the dendritic spines of somatosensory cortical layer V pyramidal neurons. A shows the micrograph of a representative segment of the proximal apical dendrite of neuron of the young adult, OHE-2W, OHE-2W+GS, OHE-8W, OHE-8W+GS, late middle age, late middle age+GS and late middle age+E2 groups, respectively. Densities of dendritic spines in these groups were analyzed in B. *, p<0.05 between the marked and young adult; #, p<0.05 between the genistein or estradiol-treated and its corresponding control, one-way ANOVA followed by Newman-Keuls test. Bar = 10 µm for all.

OHE however did not alter the dendritic arbors of the layer III and layer V somatosensory cortical pyramidal neurons of young adult females (data not shown). We therefore focused on the changes of dendritic spines following OHE and the effects of subsequent treatments. As compared to young control animals, spine densities on layer III pyramidal neurons decreased by 23–38% following OHE in comparison to that of the young adult animals (Fig. 3; F = 27.68, p<0.05 for proximal apical dendrite; F = 77.32, p<0.05 for distal apical dendrite; F = 47.87, P<0.05 for proximal basal dendrite; F = 69.78, p<0.05 for distal basal dendrite), while those on layer V pyramidal neurons were reduced by 11–35% (Fig. 4; F = 43.68, P<0.05 for proximal apical dendrite; F = 43.31, P<0.05 for distal apical dendrite; F = 9.15, P<0.05 for proximal basal dendrite; F = 18.53, p<0.05 for distal basal dendrite). There was no consistent difference between animals surviving 2 or 8 weeks following OHE (Fig. 3). Genistein treatment markedly increased the densities of spines on the four representative dendritic segments of both layer III (Fig. 3) and layer V pyramidal neurons (Fig. 4) 2 or 8 weeks following OHE. Nonetheless, spines on the proximal basal dendrites of the layer III pyramidal neurons (Fig. 3) and the distal basal dendrites of the layer V pyramidal neurons (Fig. 4) of the genistein-treated OHE-2 week animals, and also those on the distal apical and proximal basal of the layer III pyramidal neurons of genistein-treated OHE-8 week animals (Fig. 3) remained moderately fewer than those of the normal control counterparts.

**Figure 5:**
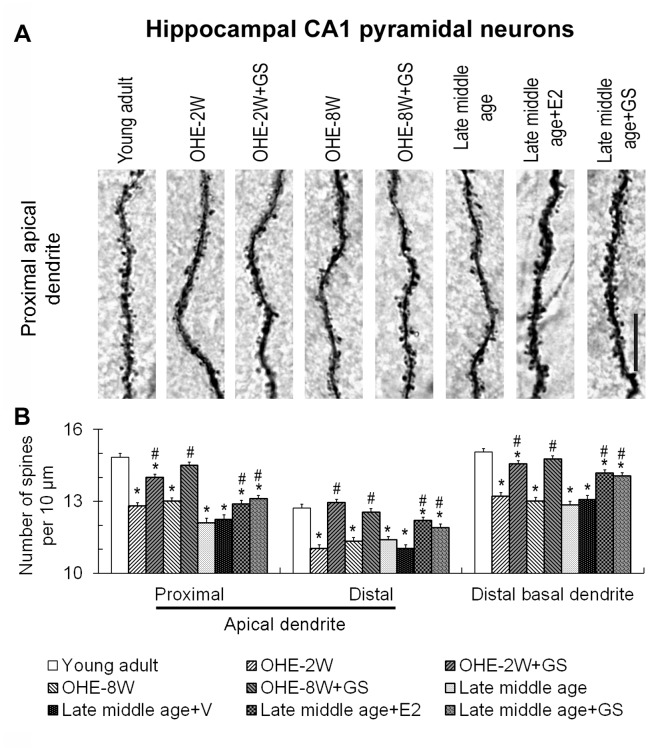
Changes in the dendritic spines of hippocampal CA1 pyramidal neurons. A shows micrographs of a representative proximal apical dendritic segment of the hippocampal CA1 pyramidal neuron of young adult, OHE-2W, OHE-2W+GS, OHE-8W, OHE-8W+GS, late middle age, late middle age+GS and late middle age+E2 groups, respectively. Please see figure 1 legend for abbreviations of experimental groups. Analyses of the spine density of these neurons were plotted in B. *, p<0.05 between the marked and young adult; #, p<0.05 between genistein or estradiol-treated and its corresponding control, one-way ANOVA followed by Newman-Keuls test. Bar = 10 µm for all.

Besides somatosensory cortical neurons, we also examined the effect on the dendritic spines of CA1 pyramidal neurons of the dorsal hippocampus because they are reported to be involved in spatial memory acquisition. Since the apical dendrites of these neurons were relatively long and their spine density changed with distance from the soma (Fig. 5, young adult), we sampled their proximal and distal segments separately. For the basal dendrite, density of spines on distal segments was measured. Results show that the densities of spines on all three segments were consistently reduced following OHE (Fig. 5; F = 19.44, p<0.05 for proximal apical dendrite; F = 12.71, p<0.05 for distal apical dendrite; F = 40.7, p<0.05 for basal dendrite) and there appears to be no consistent difference between animals surviving for 2 or 8 weeks (Fig. 5). Genistein treatment significantly increased the number of dendritic spines on all representative segments of the dendrites of the OHE-2 and 8-week animals to control levels (Fig. 5). Dendritic spines on the distal basal dendrites of the CA1 pyramidal neurons and on the proximal apical and distal basal dendrites of the CA1 neurons of the genistein-treated OHE-2-week animals nevertheless remained slightly fewer than those of the normal control counterparts (Fig. 5).

### Effects of Genistein on Late Middle Age Female Rats

Like young adults, late middle age females could learn in the spatial memory test (Fig. 1B); however, aging did compromise animals’ performance as shown in the relatively long swimming distance and the performance on the third Morris maze task day (Fig. 1B, compared the late middle age with young adult). This, however, was accompanied by a slight decrease in the animals’ swimming speed at this age (Fig. 1E, compared the late middle age with young adult, F = 9.25, p<0.01). Genistein treatment significantly improved late middle age animals’ performance as revealed by analyzing their performance on the third test day (Fig. 1B and D; F = 3.65, p<0.05 for swimming distance; F = 9.31, p<0.05 for escape time) without affecting their motor ability (Fig. 1E). To find out whether genistein was as effective as estradiol, another group of late middle age females were treated with estradiol pellet implantation and similarly tested. The results showed that genistein improved the performance of animals as effective as estradiol, i.e. it reduced the swimming distance and escape time significantly (Fig. 1B and D; F = 3.65, p<0.05 for swimming distance; F = 9.31, p<0.05 for escape time). Neither treatment changed locomotor ability (Fig. 1E).

**Figure 6:**
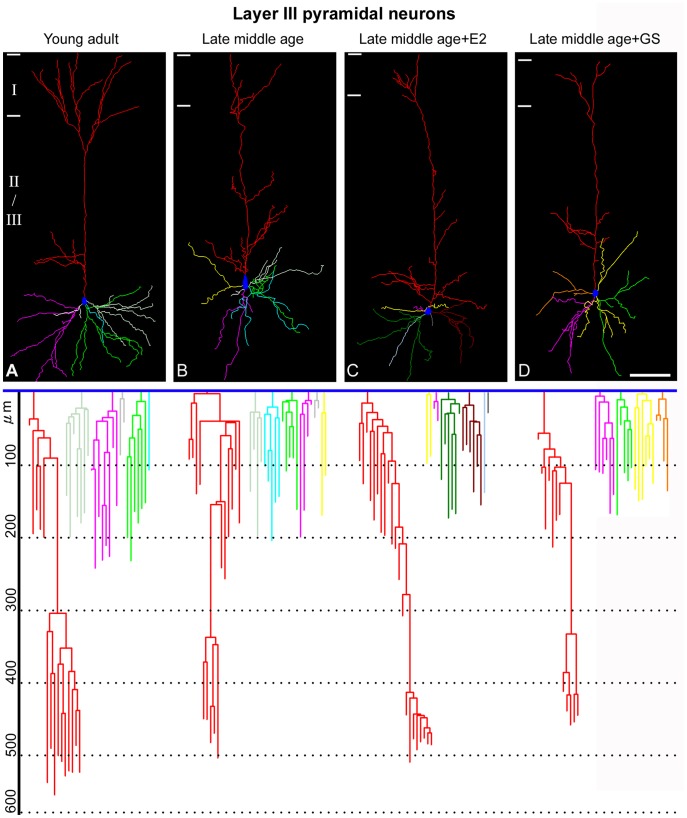
The dendritic arbors of somatosensory cortical layer III pyramidal neurons of normal and experimentally treated late middle age female rats. Representative 3-dimensionally reconstructed dendritic arbors of neurons from the young adult (A), late middle age (B), late middle age+GS (C) and late middle age+E2 (D) animals are illustrated. Roman numerals and bars to the left of each panel mark cortical laminae. The dendrogram of each neuron was shown below its dendritic arbor plot. Branches of the same dendritic trunk are shown with the same color. Bar = 100 µm for A–D.

**Figure 7:**
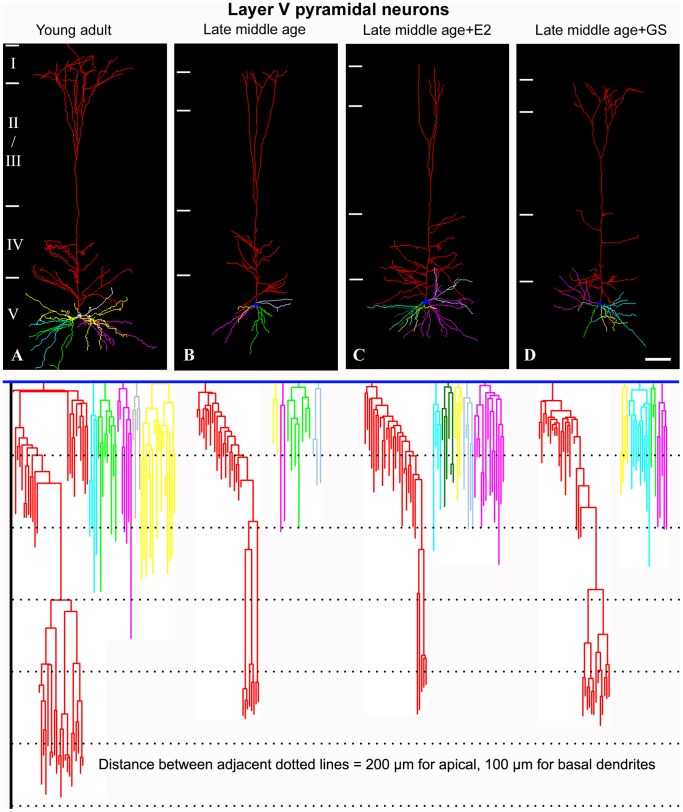
The dendritic arbors of the somatosensory cortical layer V pyramidal neuron in the somatosensory cortex of normal and experimentally treated late middle age females rats. Representative 3-dimensionally reconstructed dendritic arbor of the layer V pyramidal neurons of the young adult, late middle age, late middle age+E2 and late middle age+GS animals were illustrated in A–D respectively. Roman numerals and bars to the left of each panel mark cortical laminae. The dendrogram of each neuron was shown below. Branches of the same dendritic trunk are shown in the same color. Bar = 100 µm for A–D.

**Figure 8:**
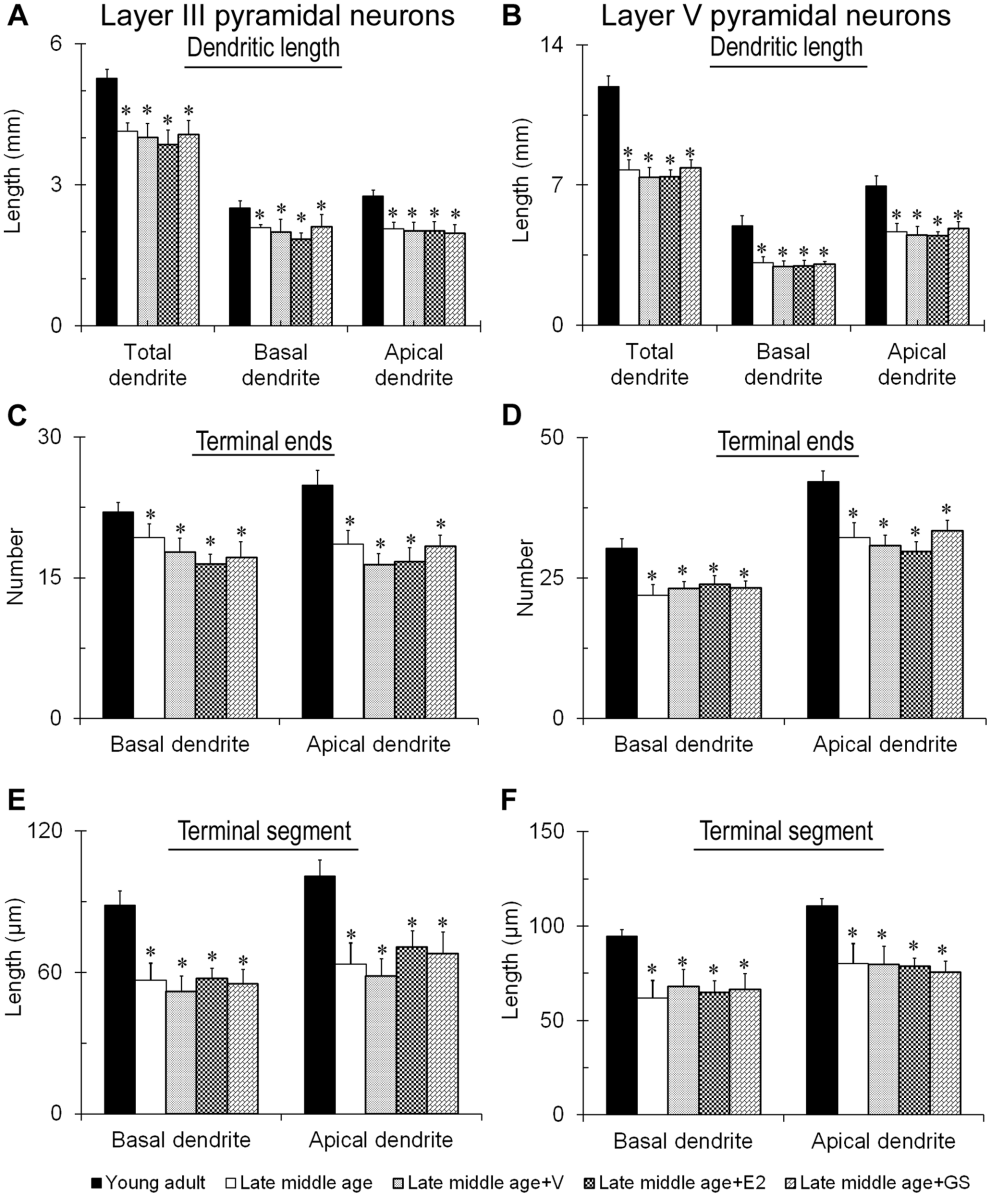
Analyses of the dendritic arbors of somatosensory cortical layer III and V pyramidal neurons of late middle age females. The total, apical and basal dendritic lengths (A and B), numbers of terminal ends of the basal and apical dendrites (C and D) and lengths of the terminal segment of the basal and apical dendrites (E and F) of the two categories of neurons were analyzed based on the 3-dimensionally reconstructed dendritic arbors with Neurolucida.

We next analyzed the dendritic arbors and spine density of the cortical pyramidal neurons in the late middle age females for aging might also affect dendritic arbors as well. Results showed that although both layer III and layer V neurons remained pyramidal in shape, their dendritic arbors were significantly reduced as compared to young adult (Figs. 6 and 7). Changes included reduction in the apparent complexity of dendrogram (Figs. 6 and 7, bottom row), total and apical and basal dendritic lengths (Fig. 8A and B; F = 5.91, p<0.05 for the basal dendritic length of layer III pyramidal neurons; F = 3.63, p<0.05 for the apical dendritic length of layer III pyramidal neurons; F = 6.47, p<0.05 for the total dendritic length of layer III pyramidal neurons; F = 9.98, p<0.05 for the basal dendritic length of layer V pyramidal neurons; F = 9.11, p<0.05 for the apical dendritic length of layer V pyramidal neurons; F = 24.22, p<0.05 for the total dendritic length of layer V pyramidal neurons), numbers of dendritic terminals (Fig. 8C and D; F = 5.89, p<0.05 for the basal terminal ends of layer III pyramidal neurons; F = 4.45, p<0.05 for the apical terminal ends of layer III pyramidal neurons; F = 3.49, p<0.05 for the basal terminal ends of layer V pyramidal neurons; F = 8.36, p<0.05 for the apical terminal ends of layer V pyramidal neurons) and mean lengths of terminal dendritic segments (Fig. 8E and F; F = 12.98, p<0.05 for the basal terminal segment length of layer III pyramidal neurons; F = 6.07, p<0.05 for the apical terminal segment length of layer III pyramidal neurons; F = 4.49, p<0.05 for the basal terminal segment length of layer V pyramidal neurons; F = 5.39, p<0.05 for the apical terminal segment length of layer V pyramidal neurons). Genistein as well as estradiol treatment did not reverse the dendritic arbors of these neurons to young adult level; all aspects of the dendritic arbors analyzed remained significantly lower than that of the young adult counterparts (Figs. 6, 7, and 8).

In addition to dendritic arbors, late middle age female rats also had reduced dendritic spines on their somatosensory cortical pyramidal neurons. Spine densities on layer III and layer V pyramidal neurons were 25–38% and 15–32% fewer than those of the young adult counterparts respectively. Genistein treatment increased the dendritic spine density on all segments of the dendrites of both neurons significantly (Figs. 3 and 4), which, however, remained noticeably less than that of the young adult counterparts in all dendritic segments analyzed (Figs. 3 and 4). To ascertain the effectiveness of genistein in comparison to estrogen, we treated another late middle age female group with estrogen. The results show that estrogen treatment restored the density of dendritic spines on these neurons to levels comparable to those promoted by genistein (Figs. 3 and 4; compared late middle age+GS with late middle age+E2), i.e., although the density of dendritic spines on these cortical neurons had recovered, it remained markedly less than those of the young counterparts.

Genistein treatment increased the dendritic spines on all segments of the dendrites of hippocampal CA1 pyramidal neurons of late middle age females too (Fig. 5). Like cortical pyramidal neurons, the restoring effects on CA1 pyramidal neurons were partial and spine density remained significantly less than those of the young adult counterparts (Fig. 5). The dendritic spine restoring effect of estradiol was also comparable to that of the genistein in hippocampal neurons (Fig. 5).

## Discussion

The major finding of this study is genistein, a phytoestrogen, treatment could restore the dendritic spines of primary somatosensory cortical and hippocampal CA1 pyramidal neurons depleted by hypogonadism. The reinstatement was close to complete in young adult females subjected to OHE but less thorough in late middle age females. Repopulation of dendritic spines on these neurons was accompanied by an improvement in spatial learning performance in Morris water maze. In addition, the present results showed that genistein was as potent as estrogen in ameliorating the loss of dendritic spines without altering blood estrogen level. Neither genistein nor estrogen was able to reinstate the dendritic arbors of somatosensory cortical pyramidal neurons of late middle age females to young adult levels.

### Blood Estrogen Levels Modulate Primary Cortical and Hippocampal Neuronal Dendritic Spine Density

There is increasing evidence that estrogen could alter the morphology of brain neurons including in the arcuate nucleus [27], lateral septum [28], ventromedial hypothalamus [29], hippocampus [30] and posterior medial amygdala [31]. In agreement with this, we recently demonstrated that estrogen affected primary somatosensory cortical neurons as well. Depleting blood estrogen in young female rats with OHE reduced dendritic spines on layer III and layer V somatosensory cortical pyramidal neurons [2], while replenishing estrogen level restored the lost dendritic spines [2]. Reduction of dendritic spines on pyramidal neurons of prefrontal cortex [5] or CA1 hippocampus [33] was also identified in aging female rats. More strikingly, estrogen was found to modulate the dendritic spines on primary somatosensory pyramidal neurons in phase with the fast estrous cycle in female rats [2] suggesting that it has an acute regulatory role in primary cortical function.

The dependence of the dendritic spines of CA1 pyramidal neurons on blood estrogen is not surprising as it has been repetitively shown [12] after the discovery that estrogen enhances the spine density of the apical dendrites of hippocampal CA1 pyramidal cells [30]. Loss of dendritic spines in hippocampal CA1 pyramidal neurons has also been demonstrated in normal aging rats and senescence-accelerated mice [32], [33]. The deterioration of spatial learning in young adult OHE and late middle age female rats is consistent with the notion that dorsal hippocampus is required for learning and memory and that the concomitant reduction of dendritic spines on their neurons could underlie this.

There are several candidate mechanisms for the modulation of dendritic spines on cortical and hippocampal neurons by estrogen. In both cerebral cortex and hippocampus, GABAergic interneurons express nuclear ERα and ERβ [34], [35]. This provides a pathway for estrogen to affect interneurons and perhaps consequently facilitate dendritic spine formation on cortical pyramidal cells. ERα is also expressed in cholinergic neurons of the basal forebrain, neocortex, and hippocampus [36], [37] suggesting an alternative of regulating cholinergic pathway to modulate cortical neuronal dendritic spine plasticity [38]. With electron microscopy, ERα and ERβ appear to have similar cellular and subcellular localization with ERβ being more extensively distributed at the extranuclear sites in the cerebral cortex and hippocampus [35], [39]–[42]. The expressions of ERα and ERβ in the cerebral cortex and hippocampus appeared to decline in middle-age and aged rats [43], [44]. Estrogen treatment was found to increase ERα expression in young adult and middle-age but not advanced aged rats [45]–[47]. This phenomenon was not seen in ERβ [46]–[48]. Recent studies had further shown that increasing hippocampal levels of ERα in middle-age rats in the absence of ovarian or exogenously administered estrogens enhanced spatial memory and increased the phosphorylation of ERK/MAPK without affecting choline acetyltransferase level or Akt’s phosphorylation [49]. Besides ERα and ERβ, our ongoing studies found that layer III and layer V pyramidal neurons of the cerebral cortex expressed high levels of the novel estrogen receptor GPR30 (unpublished data), which is an integral membrane protein of the rhodopsin-like G protein-coupled receptor family that shows high affinity for estrogen [50]. Thus, ERα and ERβ in the cortex and hippocampus and GPR30 on cortical pyramidal neuronal membrane are likely sites to mediate the posttranscriptional signaling effect of estrogen that could be further explored.

### The Effectiveness of Exogenous Estrogen in Aging Female Rats

It has been reported that the usefulness of estrogen treatment in female rats changes with age; estrogen treatments improved the spatial memory of middle-age rats [16], [51]–[54], yet its benefits declined and larger doses of estrogen were required as rats aged [55]. Some studies showed that estrogen treatment improved the spatial learning in middle-age but not advanced age rats [17], [53], [56]. This phenomenon might be related to the distribution and or changes of estrogen receptors [57]. Morphological studies showed that estrogen treatment induced synaptogenesis; increased dendritic spines and synaptic molecular markers in young adult and middle-age rats [13], [58]–[62]. The ability of estrogen to promote dendritic spine growth in rat hippocampus was found to decline with age [63]–[65]. Estrogen treatment of older animals caused hippocampal synapses to alter their NMDA receptor subunit (NR1) without changing synaptogenesis [63], [66]. Ongoing studies showed that the decreased responsiveness of hippocampal synapses to estrogen in aged animals may result from age-related decrements in ERα levels [45], [65] and alterations in LIM-kinase signaling pathways [65]. Phosphorylated-LIM kinase (pLIMK), a serine/threonine kinase [67], is activated by estrogen in young female hippocampus and plays a key role in the dynamic regulation of actin polymerization and depolymerization through cofilin phosphorylation [68]–[70]. Interestingly, another report indicates that the expression of synaptic Erβ declines with age in rats and estrogen treatment continues to enhance synaptic ERβ expression in older rats [71]. In the light of these, the expressions of ERα or Erβ in late middle age rats and their changes following genistein treatment shall be studied to explore the mechanisms of phytoestrogen’s action.

### Genistein is as Effective as Estrogen in Maintain Dendritic Spines

Genistein is a phytoestrogen that belongs to a group of plant compounds structurally and functionally similar to estrogens produced by the body, such as estradiol. Phytoestrogen has received great attention in recent years because of the potential preventive roles against chronic diseases such as hypogonadism-related cardiovascular diseases and osteoporosis and hormone-related cancers [20]–[22]. Besides GPR30, phytoestrogens preferentially bind to ERβ than ERα [72]. This implies that phytoestrogen may be more effective in areas rich in ERβ expression, including the brain, cardiovascular tissue and bone rich [73]. Thus, it’s not surprising that high dietary phytoestrogen can enhance spatial memory and increase the number of dendritic spine on hippocampal and prefrontal cortical neurons of young ovariectomized rats [74]. In the present study, we tested more specifically the effect of genistein, a major phytoestrogen, which is present in the commonly available soybean food product and has been shown to be the most efficacious phytoestrogen in human subjects and animal models using behavioral assay [19], [75]. Higher density of dendritic spines presumably reflects more excitatory synapses [2]. This and the enhancement of estrogen on NMDA receptor synaptic current [76] could have accounted for the improvement of spatial learning that we observed and consistent with earlier reports that estrogen replacement facilitates learning in rats [16], [76], [77], primates [78], [79] and humans [80], [81].

In this study, genistein also shows a dendritic spine promoting effect in the brains of the late middle age female rats. Like in surgically hypogonadal young adult females, the effects were accompanied by an improvement in animals’ spatial learning. In addition, subcutaneous genistein treatment is as potent as subcutaneous estradiol pellet implantation as assessed by the morphological and behavioral means that we adopted. Thus, phytoestrogen, especially genistein, appears to be a good alternative to estrogen for improving estrogen insufficiency syndromes in menopause and aging considering that estrogen replacement therapy risks side effects [82]–[84]. However, neither genistein nor estradiol treatment could restore the small dendritic arbors characteristic of the primary cortical neurons of aging rats back to young adult level.

### Differential Regulation of Dendritic Arbors & Spines in Aging Females

In contrast to young counterparts, dendritic spines and dendritic arbors of the layer III and layer V somatosensory cortical pyramidal neurons in the late middle age females were significantly reduced [6]. Changes in dendritic arbor include reductions of branching profuseness, total dendritic length, total dendritic terminals and length of terminal dendritic segments. Similar dendritic arbor changes were also identified in aging male rats (unpublished observation). Unlike dendritic spines, dendritic arbors of the late middle age females did not respond to estrogen or genistein treatments. However, OHE depleted blood estrogen and reduced the dendritic spines but not the dendritic arbor in young adult females. These together argue that dendritic arbors are modulated by mechanisms different from that regulate dendritic spines. In this regard, dendritic arbors could be highly susceptible to the availability of trophic factors, which are critical to neurite outgrowth and branching and are expected to decrease with ages [85], [86]. On the other hand, dendritic spines are likely to be more dependent on synaptic activities and factors such as gonadal hormones [2], [30], [87], cholinergic innervation [88], environment changes [1], stress [26], and even mechanical force [7], [89]. The incomplete rescuing effects of both genistein and estradiol on the dendritic spines of hippocampal and primary cortical pyramidal neurons in the late middle age females further supports the argument that blood estrogen is not the sole determinant of dendritic spine density. Interactions between different factors that regulate dendritic spines, especially in aging animal, remain to be explored.

In conclusion, late middle age and OHE-young adult females showed reduced dendritic spines on their primary somatosensory cortical pyramidal and hippocampal pyramidal neurons. These animals at the same time showed poorer spatial learning. In young adult females, genistein treatment almost completely reversed the morphological and spatial memory changes caused by surgical estropause. In late middle age females, genistein although partial and effectively reversed the changes associated with aging. Thus, genistein has a great potential as a therapeutic drug that works at the central neuronal level to rescue the cognitive ability lost to aging or menopause.

## Supporting Information

Figure S1 **Three-dimensional reconstruction of the dendritic arbor of pyramidal neurons.** A layer V pyramidal neuron of normal aging rats was 3-dimensionally reconstructed with Neurolucida. The 350-µm thick brain slice used for intracellular dye injection was prepared with vibratome and treated with DAPI solution to visualize cell nuclei for the selection of cell under a fluorescence microscope with 20X long-working-distance lens (A). Selected cell was filled with Lucifer yellow with constant negative current. Complete filling of layer V pyramidal neuron took about 10–15 min. The brain slice was then postfixed and cryosectioned into 60-µm-thick serial sections for subsequent immunoconversion (B). All segments of the neuron’s dendritic arbor were stereologically reconstructed through the serial sections sequentially with Neurolucida (C). Strung all dendritic segments of each section together revealed the whole neuron in 3-dimensional space (D). Bar = 100 µm in A–D.
(TIF)
