# Supplementary figures and images for: Genistein Partly Eases Aging and Estropause-Induced Primary Cortical Neuronal Changes in Rats
Source: PLoS One. 2014 Feb 25;9(2):e89819. doi: 10.1371/journal.pone.0089819 (PMC3934964; doi:10.1371/journal.pone.0089819)

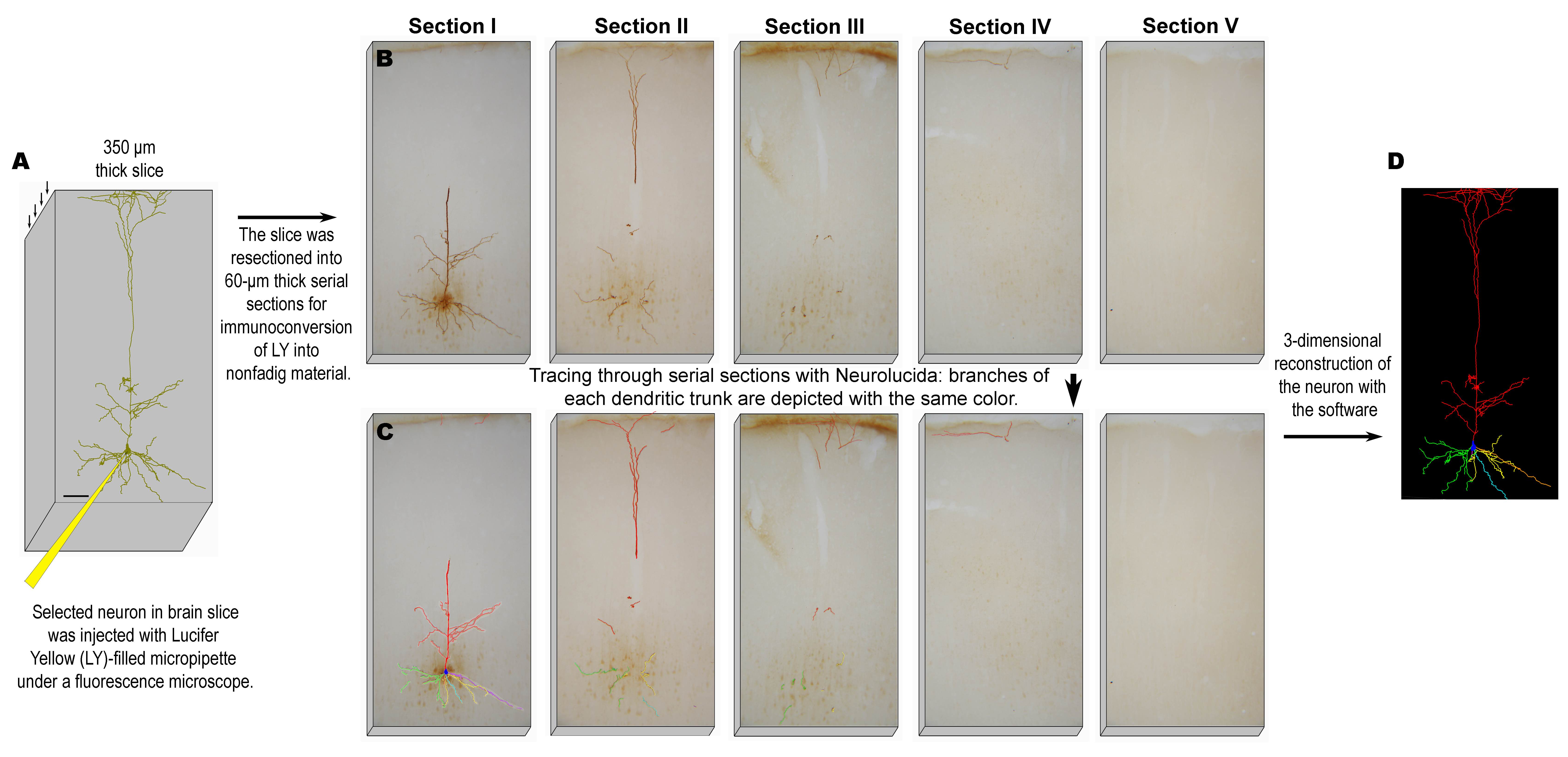

Supplement: Figure S1 — Three-dimensional reconstruction of the dendritic arbor of pyramidal neurons. A layer V pyramidal neuron of normal aging rats was 3-dimensionally reconstructed with Neurolucida. The 350-µm thick brain slice used for intracellular dye injection was prepared with vibratome and treated with DAPI solution to visualize cell nuclei for the selection of cell under a fluorescence microscope with 20X long-working-distance lens (A). Selected cell was filled with Lucifer yellow with constant negative current. Complete filling of layer V pyramidal neuron took about 10–15 min. The brain slice was then postfixed and cryosectioned into 60-µm-thick serial sections for subsequent immunoconversion (B). All segments of the neuron’s dendritic arbor were stereologically reconstructed through the serial sections sequentially with Neurolucida (C). Strung all dendritic segments of each section together revealed the whole neuron in 3-dimensional space (D). Bar = 100 µm in A–D. (TIF) [file pone.0089819.s001.tif]
